# A comparison of global end-diastolic volume (GEDI) and central venous pressure (CVP) as parameter for volumen assessment in patients during major liver resections

**DOI:** 10.1186/2197-425X-3-S1-A216

**Published:** 2015-10-01

**Authors:** FJ Redondo Calvo, N Bejarano, D Padilla, P Villarejo, V Baladrón, R Villazalo, AS Yuste, P Arenas

**Affiliations:** Facultad Medicina Ciudad Real. Hospital General Universitario Ciudad Real, Ciudad Real, Spain; Hospital de Santa Barbara, Puertollano Ciudad Real, Spain; Hospital Universitario Ciudad Real, Ciudad Real, Spain

## Goal of Study

The aim of our study was to evaluate the predictive value of CVP with regard GEDI, and correlate these parameters to cardiac Index (CI) and extravascular lung water index (EVLWI).

## Methods

Prospective study. Surgical intensive care unit, university hospital.

Patients and interventions: 89 hemodynamic measurements using the PiCCO (Pulsion Medical System, Germany) were performed in 18 patients during major liver resection

## Results

Mean CVP (8,23 +/- 3,12 mmHg) was normal, whereas mean GEDI (615,2 +/- 135,44 mL/m2) was decreased. Thirty-one CVP measurements were elevated despite simultaneous GEDI levels indicating a normal or decreased preload. Sensitivity, specificity, positive predictive value, and negative predictive value of CVP with regard to volume depletion (GEDI < 650) were 6,28 (0-12,77. CI 95%), 100 (97,86-100, CI 95%, 43, 2 (28,99-50,82, CI 95%) respectively. CVP did not correlate to GEDI (r = -0,065, p = 0,32), CI (r = 0,23, p = 0,176) and EVLWI (extravascular lung water index) (r = -0,05, p= 0,49). GEDI significantly correlated to CI (r = -0,24, p < 0,01) and VVS (r = -0,39, p < 0,01).

## Conclusions

Volume depletion according to GEDI was found in more than half the patients. The predictive values of CVP with regard to volume depletion were low GEDI and its changes significantly correlated to CI and its changes, which was not observed for CVP. Therefore, GEDI appears to be more appropriate for volume management during major liver resections with the aim to avoid intraoperative bleeding and transfusion.

## Grant Acknowledgment

We express our gratitude to Mutua Madrileña Foundation (Madrid, Spain) for its grant collaboration by without which this work could not have been completed.
